# Tunable exchange bias in dilute magnetic alloys – chiral spin glasses

**DOI:** 10.1038/srep19964

**Published:** 2016-01-28

**Authors:** Matthias Hudl, Roland Mathieu, Per Nordblad

**Affiliations:** 1 Stockholm University, Department of Physics, Chemical Physics, SE-106 91 Stockholm, Sweden; 2 Uppsala University, Department of Engineering Sciences, Solid State Physics, Box 534, SE751 21 Uppsala, Sweden

## Abstract

A unidirectional anisotropy appears in field cooled samples of dilute magnetic alloys at temperatures well below the cusp temperature of the zero field cooled magnetization curve. Magnetization measurements on a Cu(13.5 at% Mn) sample show that this anisotropy is essentially temperature independent and acts on a temperature dependent excess magnetization, ΔM. The anisotropy can be partially or fully transferred from being locked to the direction of the cooling field at lower fields to becoming locked to the direction of ΔM at larger fields, thus instead appearing as a uniaxial anisotropy. This introduces a deceiving division of the anisotropy into a superposition of a unidirectional and a uniaxial part. This two faced nature of the anisotropy has been empirically scrutinized and concluded to originate from one and the same exchange mechanism: the Dzyaloshinsky-Moriya interaction.

The unidirectional anisotropy (E_ud_) causing exchange biased hysteresis loops in dilute magnetic alloys appears in field cooled samples at temperatures well below the cusp temperature of the zero field cooled magnetization curve^1^. This anisotropy is found to have very specific characteristics: It is essentially temperature independent and acts on a temperature dependent excess magnetization (ΔM); the direction of the anisotropy field is set by the direction of the applied cooling field. The anisotropy can be partially or fully transferred from being locked to the direction of the cooling field at lower fields to becoming locked to the direction of ΔM at larger fields, thus appearing as a uniaxial anisotropy. This introduces a deceiving division of the anisotropy into a superposition of a unidirectional and a uniaxial part^2^. In this report, this two faced nature of the anisotropy is empirically scrutinized and concluded to originate from one and the same exchange mechanism: the Dzyaloshinsky-Moriya interaction^3^,^4^.

Exchange bias was first observed some 60 years ago in a field cooled system of cobalt particles with a shell of cobaltous oxide-“A new type of magnetic anisotropy has been discovered which is best described as an exchange anisotropy”-5,6. A similar type of anisotropy, causing shifted hysteresis loops, occurs in certain dilute magnetic alloys, and has been extensively investigated in Cu(Mn) alloys of different manganese concentration^7^,^8^,^9^,^10^,^11^,^12^,^13^. Atomic short range order^14^ and induced inhomogeneity may affect the hysteresis behavior of Cu(Mn) alloys as demonstrated by Monod *et al.* in Figure 8 of Ref. [10] by measurements on a cold worked sample. Such defects are revealed by severe broadening of the low field maximum of the zero field cooled (ZFC) magnetization vs. temperature curve and enhanced temperature for the onset of irreversibility between the ZFC and field cooled (FC) magnetization curves^15^. Exchange bias also occurs in bilayers of a ferromagnetic and an antiferromagnetic material and such structures have contributed to the continuously increased storage capacity in magnetic hard discs; being used as key components of both the read head and the storage medium. However, the physical mechanisms behind exchange bias are yet of unsettled origin and remain a scientific controversy^16^. Of special interest in the context of the current investigation is the exchange bias effect reported on a bilayer of spin glass, Cu(Mn 6 at%), and cobalt layers^17^.

Figure 1(a) shows field cooled (FC) hysteresis loops of our Cu(13.5 at% Mn) (hereafter referred to as CuMn) sample measured in different cooling fields, H_FC_ at the temperature T_m_ = 5 K or reduced temperature T_m_/T_g_ = 0.09 (the spin glass temperature of the sample is T_g_ = 57 K^18^). All hysteresis loops are measured in-between the maximum fields ±H_FC_. Three characterizing parameters can be extracted from these curves: An excess moment, ΔM, a first switch field H_sw1_ and a second switch field H_sw2_ as defined in Fig. 1(b). The variation of these parameters with the magnitude of the cooling field is plotted in the insets of Fig. 1(a). Figure 1(b) shows a full hysteresis loop of the CuMn sample measured up to a maximum field of 14 T. The initial branch of the hysteresis loop has a characteristic S-shape without sudden magnetization jumps. The first decreasing branch of the hysteresis loop shows a sharp jump, defining ΔM, at the switch field, H_sw1_, and on the following field increasing branch of the hysteresis loop, a magnetization reversal of the same magnitude ΔM occurs in the opposite direction at the switch field H_sw2_. As is seen in Fig. 1(a), ΔM and H_sw1_ are both essentially independent of the cooling field strength in the considered field range (1 to 9 T). H_sw2_, on the other hand, starts out from a value rather close to H_sw1_ and continuously increases with increasing H_FC_ to asymptotically approach the value -H_sw1_ at very high fields. The three parameters are weakly dependent on repeated hysteresis measurements (training) as long as the maximum field of the loops does not exceed the initial cooling field. However, if the maximum field, H_max_ of the hysteresis measurement is increased, H_sw2_ shifts to the same value as observed after field cooling in H_max_, whereas ΔM and H_sw1_ remain essentially unaffected by the field increase. Using the product of ΔM and H_sw1_ one can derive a measure of the anisotropy energy related to the switching of the excess magnetization ΔM. This anisotropy, E_udT_ ∝ ΔMH_sw1_, is independent of the magnitude of the cooling field. Looking at the field dependence of H_sw2_, one observes that at low cooling fields, the anisotropy of the sample is near unidirectional (H_sw1_ ~ H_sw2_), and that with increasing cooling field it attains the mentioned mixed unidirectional/uniaxial character^2^ and finally striving towards only a uniaxial character at very high fields. Figure 1(c) shows hysteresis loops after field cooling in +5 T and –5 T, and Fig. 1(d) shows hysteresis loops measured after zero field cooling (ZFC) and first applying the field up to 5 T in subsequently the positive and the negative direction. Remarkably, both the FC and the ZFC protocols reveal similarly exchanged biased (H_eb_ = (H_sw1_ +H_sw2_)/2) hysteresis loops.

The field controlled two-faced character of the anisotropy: unidirectional and uniaxial, may be explained by the exchange mechanism giving rise to anisotropy in a Heisenberg spin system: the Dzyaloshinsky-Moriya interaction (DMI)^3^,^4^ and the chirality^19^ of the local spin structure (see also Levy *et al.*^2^ and Staunton *et al.*^20^,^21^). Only a weak cooling field introduces one polarity of the chiral spin structures yielding unidirectional anisotropy along the field direction. This anisotropy acts on the excess magnetization, which switches when the applied magnetic field exceeds H_sw1_. This field remains independent of the strength of the cooling field. However, on field reversal, the switching field H_sw2_ is dependent on the cooling field. This behavior requires that polarity of the chirality of parts of the local spin structure switches with the excess magnetization instead of only being confined to the original cooling field direction and that the fraction of spin chirality following the switching of the excess moment is set by the magnitude of the cooling field. Using this two-faced picture of the local spin structure (locked to the cooling field direction at low fields and locked to the switching excess magnetization at high fields), only the DMI is required to explain the observed behavior and tuning of the exchange bias. At constant low temperature, the only way to change the partition between these two fractions is to apply a higher maximum field than the employed cooling field. The magnitudes of the two fractions of the DMI can be experimentally derived using the postulation that: E_udT_ = E_udS_ +E_udM_, where E_udS_ denotes the part of the anisotropy that remains locked to the direction of the cooling field and E_udM_ the part of the anisotropy that is locked to the direction of the excess moment. The two parts are then be derived from the experimental parameters through:


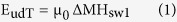



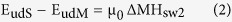


Figure 2(a) shows the hysteresis behavior after field cooling in fields varying from 0 to 5 T and measuring hysteresis loops in fields from +H_FC_ to −H_FC_. From these curves the detailed behavior of the field dependence of the characterizing parameters, including the low field region between 0 and 1 T where ΔM remains unsaturated, is derived. In Fig. 2(b), H_sw1_ and H_sw2_ are plotted versus the cooling field strength and in Fig. 2(c) the corresponding dependence of ΔM is shown. From these data, the related energy measures can be derived and these are plotted in Fig. 2(d,e) showing μ_0_ΔMH_sw1_ (E_udT_) and μ_0_ΔMH_sw2_ versus cooling field in Fig. 2(d) and the unidirectional energies E_udS_ and E_udM_ in Fig. 2(e). It is worth noting that the magnitude of the unidirectional anisotropy appears independent of cooling field strength, confirming that already an infinitesimally small cooling field is capable of aligning the polarity of the chirality in the field direction.

The temperature dependence of the hysteresis loops measured after field cooling in 1 T are shown in Fig. 3(a) and the derived parameters in Fig. 3(b,c). It is remarkable that the derived magnitude of the anisotropy energy E_udT_ also appears temperature independent. Noteworthy is also that a larger and larger part of the anisotropy becomes locked to the excess magnetization with increasing temperature. At higher temperatures than 25 K, the collective excess moment ΔM has already relaxed to zero on the time scale of these hysteresis experiments.

Our results imply that a rigid spin structure defining the anisotropy is imprinted in the spin glass on cooling to low temperatures and that only a weak field is required to establish the full strength of the unidirectional anisotropy (E_udT_) along the direction of the applied field. The only way to alter this structure and the associated anisotropy at constant temperature is to apply a larger field (H_max_) than the original cooling field. The effect of H_max_ is then to imprint a compensating unidirectional anisotropy that switches direction with ΔM and yields an anisotropy field that is controlled by H_max_. It is in this context important that in a zero field cooled sample a large field is required to establish any anisotropy and excess magnetization and that the full strength of the anisotropy is only achieved at field larger than the field where the thermoremnant magnetization (TRM) and isothermal remnant magnetization (IRM) coalesce^22^. In this connection it is worth noting that the existence of exchange bias after zero field cooling in Heisenberg spin glasses is uncharacteristic to most other exchange bias systems, however, large exchange bias has been observed by A. K. Nayak *et al.*^23^ after zero field cooling of a Mn_2_PtGa Heusler alloy. This exchange bias is assigned to induced exchange coupling between ferromagnetic clusters dispersed in a ferrimagnetic matrix when the sample is first magnetized at low temperature. Thus, a very different mechanism from the one discussed here for Heisenberg spin glasses.

The possibility to imprint a rigid spin structure in spin glasses that withstands significant perturbations is also manifested in low-field experiments on ageing-related phenomena showing memory of an aged spin structure at a specific temperature in spite of an apparent rejuvenation on temperature perturbations^24^,^25^,^26^. Two recent reviews of J. A. Mydosh^27^ and H. Kawamura and T. Taniguchi^28^ comprehensively recall the remarkable physical properties of spin glass systems including discussions on the importance of the chirality in Heisenberg systems.

## Methods

The sample is a polycrystalline cylinder of Cu(13.5 at% Mn) of height 5 mm and diameter 2 mm with a spin glass temperature of 57 K^18^. The material is prepared by the drop synthesis method^29^. The global homogeneity and composition have been ascertained by EDS (Energy Dispersive Spectroscopy) measurements on different spots of the surface area of the sample and by the sharpness of the spin glass transition at low applied magnetic fields. Fig. S1 (in the supplementary information) shows the temperature dependence of the ZFC and FC susceptibility (M/H) of the sample at different applied fields. The experiments are performed in a Quantum Design MPMS XL SQUID magnetometer (5T) and two different PPMS VSM systems (9 T and 14 T). All hysteresis measurements are performed after cooling the sample from a reference temperature, T_REF_ = 70 K.

## Additional Information

**How to cite this article**: Hudl, M. *et al.* Tunable exchange bias in dilute magnetic alloys – chiral spin glasses. *Sci. Rep.* **6**, 19964; doi: 10.1038/srep19964 (2016).

## Supplementary Material

Supplementary Information

## Figures and Tables

**Figure 1:**
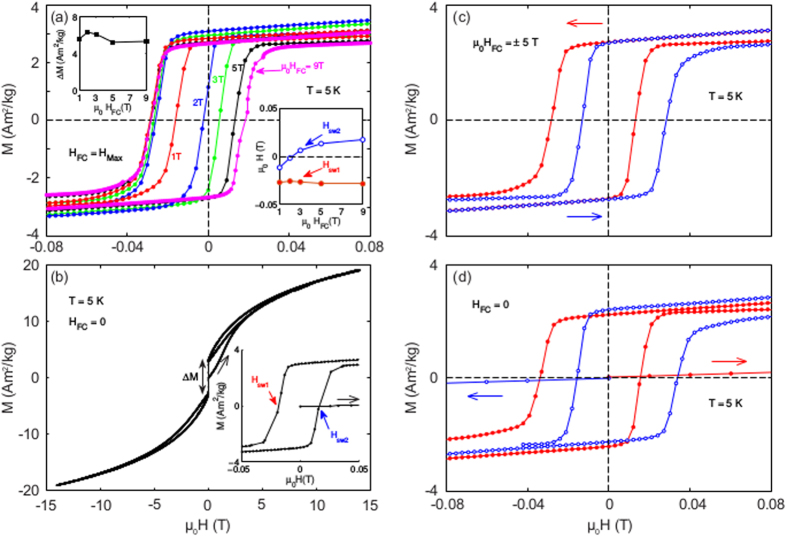
Magnetic hysteresis and tunable exchange bias. (**a**) The central part of M vs. H loops after cooling the sample in H_FC_ to 5 K, and measure the hysteresis loop on decreasing the field to – H_FC_ and back to +H_FC_. The insets show the field dependence of the parameter H_sw1_, H_sw2_, (right) and ΔM (left) in the H_FC_ range 1 to 9 T. (**b**) Full hysteresis loops measured at 5 K from 0 to 14, 14 to −14 and then back +14 T. The inset shows the central part of the loop and the fields H_sw1_ and H_sw2_ are marked. The jump and definition of the excess magnetization, ΔM, is indicated in the main frame. (**c**,**d**) The central part of the hysteresis loops measured after field cooling (**c**) and zero field cooling (**d**) in fields up to 5 T at 5 K. The arrows indicate the initial field sweep direction. (Red loops- positive initial fields, blue loops - negative initial fields).

**Figure 2:**
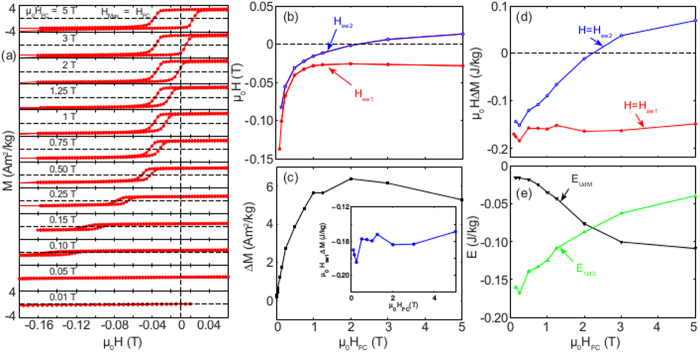
FC Hysteresis loops at different fields. (**a**) The central part of M vs H loops measured at 5 K after cooling in H_FC_ (of strength indicated in the figure) and measured in-between ±H_FC_. When H_FC_  < 0.15 T; the loops have been recorded up to fields high enough to reach H_sw1_ when measurable. The scale on the magnetization axes is always drawn between ± 4 Am^2^/kg. (**b**) Anisotropy fields and excess magnetization. (**c**) Cooling field dependence of H_sw1_ and H_sw2_ (upper panel), and ΔM (lower panel) derived from the M vs. H curves in (**a**). The inset shows the field dependence of the corresponding anisotropy energy μ_0_ΔMH_sw1_. (**d**,**e**) The energy μ_0_ΔMH, with H = H_sw1_ and H_sw2_ (**d**), and the energies E_udS_ and E_udM_ (**e**) as a function of the cooling field μ_0_H_FC_.

**Figure 3:**
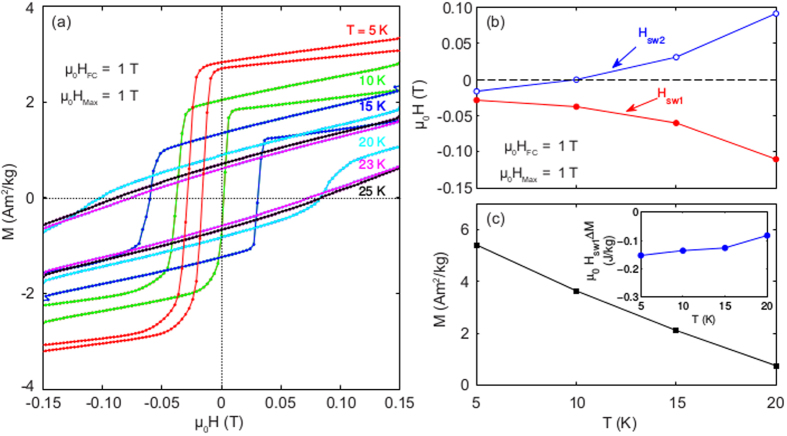
Temperature dependence of the hysteresis. (**a**) Temperature dependence of the central part of hysteresis loops measured after field cooling in H_FC_ = 1 T and recording the loops up to H_max_ = ±1T. (**b**) Temperature dependence of H_sw1_ and H_sw2_ derived from (**a**). (**c**) The temperature dependence of ΔM and the inset shows the derived anisotropy μ_0_ΔMH_sw1_ vs. temperature.
