# Supplementary material for: Tunable exchange bias in dilute magnetic alloys – chiral spin glasses
Source: Sci Rep. 2016 Jan 28;6:19964. doi: 10.1038/srep19964 (PMC4730205; doi:10.1038/srep19964)
Supplement: Supplementary Information [file srep19964-s1.pdf]

Supplementary:

Tunable exchange bias in dilute magnetic alloys – chiral spin glasses.

Matthias Hudl[§], Roland Mathieu[#] & Per Nordblad[#*]

[§]Stockholm University, Department of Physics, Chemical Physics, SE-106 91 Stockholm, Sweden.

[#] Uppsala University, Department of Engineering Sciences, Solid State Physics, Box 534, SE751 21 Uppsala, Sweden.

[*]per.nordblad@angstrom.uu.se

The temperature dependence of the dc-susceptibility, M/H, in zero field cooled (ZFC) and field cooled (FC) protocols and different applied fields of the Cu(13.5 at% Mn) sample is shown in Fig. S1. Some spin glass features are worth noting: $M_{FC}(T)/H$ is always suppressed by the magnetic field whereas the $M_{ZFC}(T)/H$ is enhanced at higher fields and temperatures below the bifurcation temperature of the FC and ZFC curves. The field dependence of the bifurcation temperature can be used to construct a constant relaxation time contour in the H-T plane. Figure S2 shows a schematic HT-diagram with a constant relaxation time contour: This line separates a low temperature region of magnetic irreversibility from a high temperature with reversible magnetization behavior on the time scale (10-100 s) of the ZFC experiments. The arrows in the figure illustrate different experimental protocols: field cooling and application of fields remaining in the irreversible region and into the reversible region at higher fields and temperatures.

Referring to these two figures, the reported experiments can be divided into three categories:

1. The data in Fig. 1 of the main text is derived from M vs. H experiments at 5 K after field cooling (or zero field cooling) at magnetic fields ($1 \leq H_{FC} \leq 14$ T) that are high enough to yield a saturated excess magnetization ΔM; i.e. at H_{FC} that are found close to the constant relaxation time contour (or irreversibility line) (cf. Figs. S1 and S2) and well into the reversible part of the HT-phase diagram (H_{Rev}).
2. The experiments in Fig. 2 of the main text are performed after field cooling from T_{ref} to T_M=5 K in fields ranging between 0.01 and 5 T; i.e. at fields in the irreversible regime of the HT-phase diagram and well into the reversible region. In this region ΔM is built up from being very small at 0.01 T to reaching the saturated value at the higher fields.
3. The data in Fig. 3 is measured after field cooling in 1 T and recording M versus H at different temperatures; i.e. the cooling field falls further and further above the irreversibility line with increasing temperature. From these results, one learns that the unidirectional anisotropy, E_{udT}, is created already when the irreversibility line is crossed during cooling, whereas measurable excess magnetization ΔM only occurs at temperatures below about 20 K. It is also seen from the M vs. H curves at 23 and 25 K that a non-exchanged biased thermoremanent magnetization, TRM, remains at higher temperatures.

Figure captions:

Figure S1 the zero field cooled and field cooled apparent susceptibility (M_{ZFC}/H and M_{FC}/H) plotted vs temperature at different applied fields as indicated in the figure.

Figure S2 a schematic HT-phase diagram indicating a constant relaxation time contour. The arrows and the temperature and field labels refer to the experimental protocols employed in the reported studies of exchange bias in CuMn.

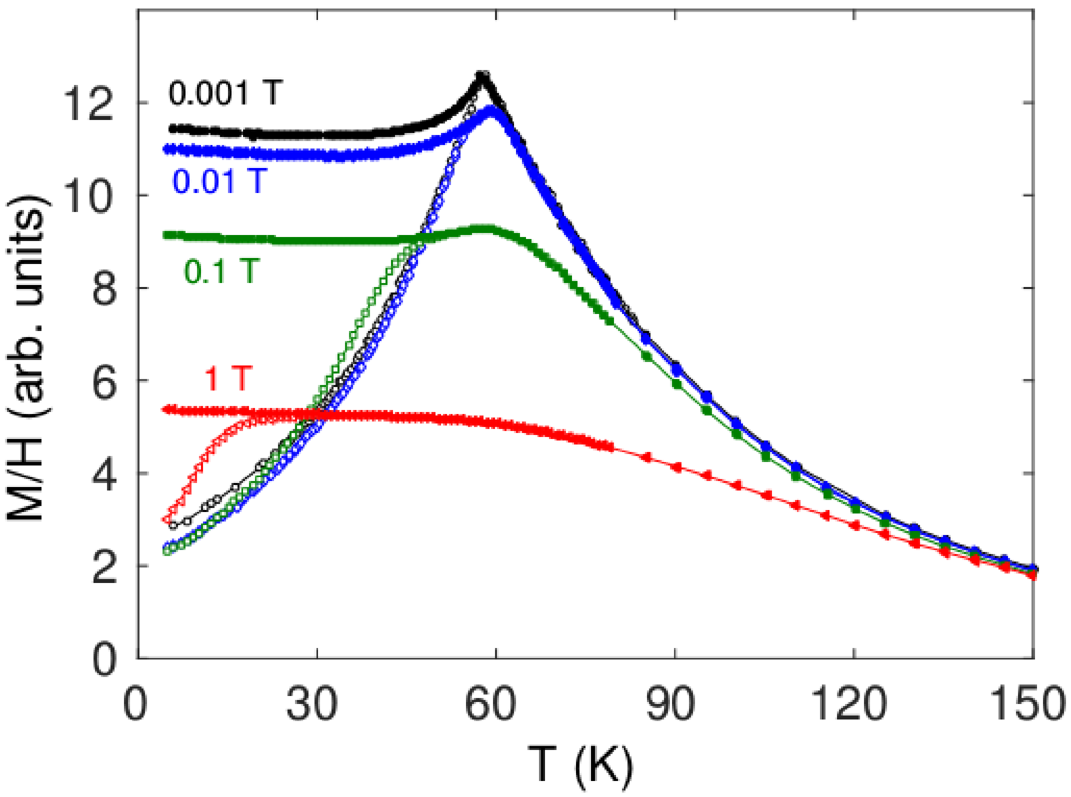

Figure S1

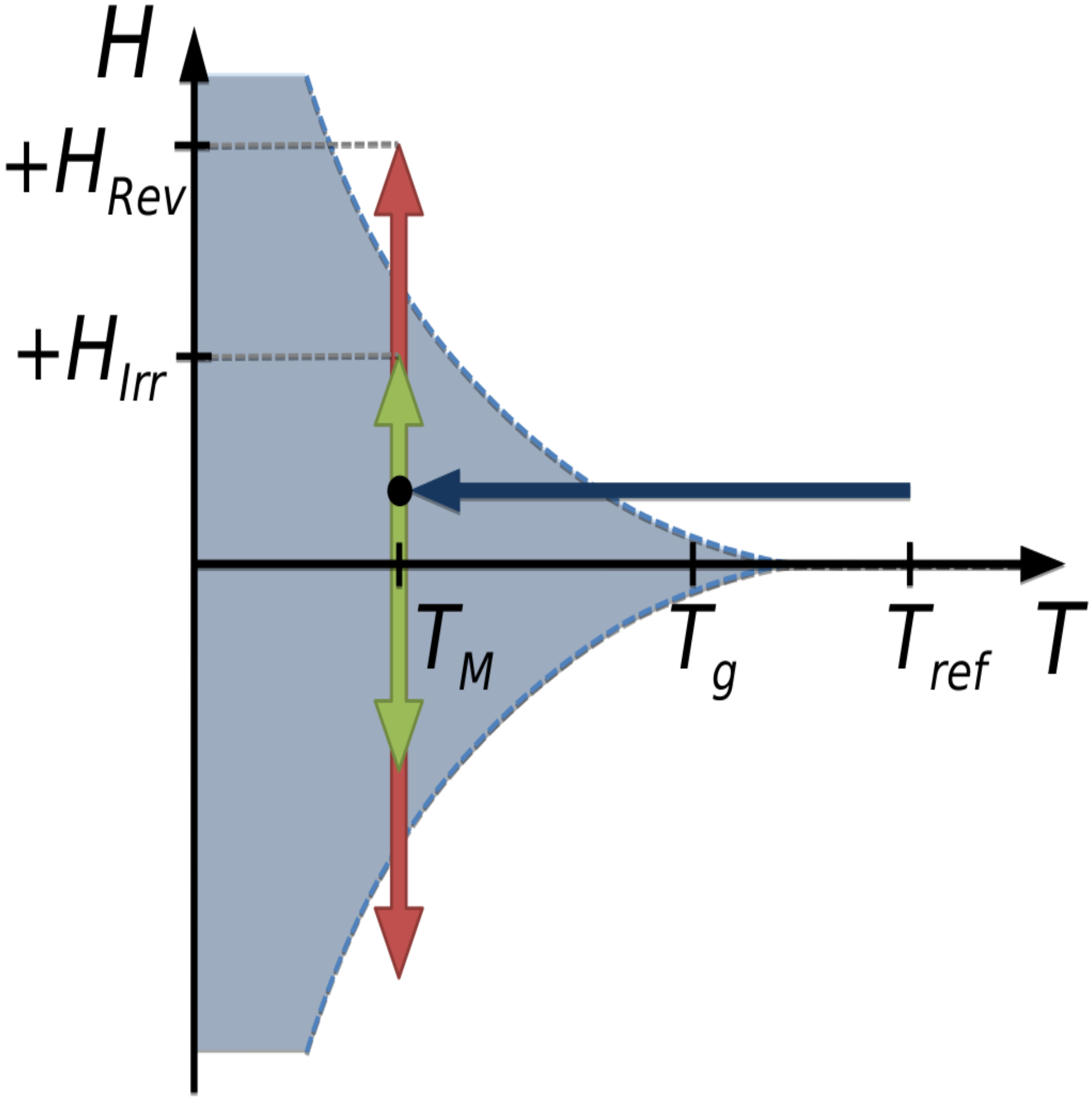

Figure S2
